# *De Novo* Sequencing and Analysis of the Safflower Transcriptome to Discover Putative Genes Associated with Safflor Yellow in *Carthamus tinctorius* L.

**DOI:** 10.3390/ijms161025657

**Published:** 2015-10-26

**Authors:** Xiuming Liu, Yuanyuan Dong, Na Yao, Yu Zhang, Nan Wang, Xiyan Cui, Xiaowei Li, Yanfang Wang, Fawei Wang, Jing Yang, Lili Guan, Linna Du, Haiyan Li, Xiaokun Li

**Affiliations:** 1 Ministry of Education Engineering Research Center of Bioreactor and Pharmaceutical Development, Jilin Agricultural University, Changchun 130118, Jilin, China; E-Mails: xiuming1211@163.com (X.L.); yydong@aliyun.com (Y.D.); nayao@163.com (N.Y.); wangnanlunwen@126.com (N.W.); xiaoweili1206@163.com (X.L.); nifengcao_2000@163.com (Y.W.); fw-1980@163.com (F.W.); yangjing5122010@163.com (J.Y.); guanll2004@163.com (L.G.); dulinna0918@163.com (L.D.); 2 College of Life Sciences, Jilin Agricultural University, Changchun 130118, Jilin, China; E-Mails: jauzhangyu@163.com (Y.Z.); cuixiyan2005@163.com (X.C.)

**Keywords:** *Carthamus tinctorius* L., 454 sequencing, flavonoid biosynthesis, safflor yellow, transcriptome

## Abstract

Safflower (*Carthamus tinctorius* L.), an important traditional Chinese medicine, is cultured widely for its pharmacological effects, but little is known regarding the genes related to the metabolic regulation of the safflower’s yellow pigment. To investigate genes related to safflor yellow biosynthesis, 454 pyrosequencing of flower RNA at different developmental stages was performed, generating large databases.In this study, we analyzed 454 sequencing data from different flowering stages in safflower. In total, 1,151,324 raw reads and 1,140,594 clean reads were produced, which were assembled into 51,591 unigenes with an average length of 679 bp and a maximum length of 5109 bp. Among the unigenes, 40,139 were in the early group, 39,768 were obtained from the full group and 28,316 were detected in both samples. With the threshold of “log2 ratio ≥ 1”, there were 34,464 differentially expressed genes, of which 18,043 were up-regulated and 16,421 were down-regulated in the early flower library. Based on the annotations of the unigenes, 281 pathways were predicted. We selected 12 putative genes and analyzed their expression levels using quantitative real time-PCR. The results were consistent with the 454 sequencing results. In addition, the expression of chalcone synthase, chalcone isomerase and anthocyanidin synthase, which are involved in safflor yellow biosynthesis and safflower yellow pigment (SYP) content, were analyzed in different flowering periods, indicating that their expression levels were related to SYP synthesis. Moreover, to further confirm the results of the 454 pyrosequencing, full-length cDNA of chalcone isomerase (CHI) and anthocyanidin synthase (ANS) were cloned from safflower petal by RACE (Rapid-amplification of cDNA ends) method according to fragment of the transcriptome.

## 1. Introduction

Safflower (*Carthamus tinctorius* L.), is a widely used herbal plant in the family *Compositae*, which are cultured in many countries worldwide. In China, safflower plays an important role in meals, dye and traditional medicine [1,2]. Safflower flowers have important pharmacological effects, such as promoting blood circulation to remove blood stasis and alleviate pain [3,4]. The chemical components of safflower are diverse, including flavonoids [5] such as safflor yellow, alkaloids and fatty acids [6], polysaccharide and others.

Safflower yellow pigments (SYPs), which are isolated from safflower petals, as flavonoid compounds, have been extensively applied in many fields, including as a medicine and natural food colorant. Hydroxysafflor Yellow A (HSYA), is the major active component of the flower and has potent and important antioxidative effects *in vitro* [7,8], an enormous antagonistic impact on platelet activating factor receptor [9] and vascular dementia [10], and an inhibitory effect on platelet aggregation, tumor angiogenesis, thrombosis and oxidative stress [11,12]. In China, HSYA has been used clinically in the cerebrospinal fluid of patients with traumatic brain injuries [13]. Related research [14] showed that the maximal inhibitory action against the proliferation of 3T3-L1 cells was 0.1 mg/L HSYA over 72 h. Although HSYA is the flavonoid component of the safflower yellow pigments, the relationship between the HSYA composition in safflower petals and the key enzymes catalyzing flavonoid biosynthesis have not been demonstrated.

With the development of high-throughput next-generation sequencing (NGS) technology, RNA sequencing (RNA-Seq), as an effective alternative technology, has been applied extensively to animals, plants and microorganisms, providing novel candidate genes, and validating and refining gene models for metabolic pathways [15,16,17]. The mass of transcript sequences obtained from RNA-Seq has led to the annotation of functional genes included in biological processes [18,19]. Recently, some sequencing by synthesis (SBS) methods [20] have started using NGS platforms, such as the Roche/454 Genome Sequencer FLX Instrument, the Illumina Genome Analyzer, and the ABI SOLiD System. Of these, the 454 pyrosequencing platform provides a remarkably effective sequencing technology to research the transcriptomes of unknown plant genomes [21,22].

Because of the important pharmacological effects of safflower petals, the aims of this study were to investigate the *de novo* transcriptome in the early and full flowering stages of safflower using the 454 pyrosequencing platform. More importantly, on gene expression levels and identifications, functional annotations, and functional genomic studies could be explored using these transcripts. Based on the sequencing, the *de novo* assembly and characterization of the transcriptome of safflower was performed, and key genes involved in flavonoid biosynthesis were isolated, which established a biotechnological platform for further research on safflower.

## 2. Results

### 2.1. Preparation, Sequencing and de Novo Assembly of the Flower Transcriptome

**Figure 1:**
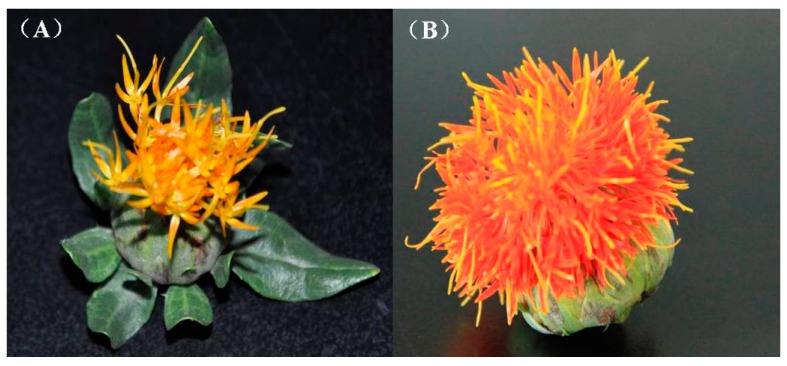
Safflower flower phenotypes in different flowering stages. (**A**) early stage after flowering; and (**B**) full stage after flowering.

To comprehensively cover the optimal flowering period, which had a higher level of expression for a particular condition, total RNA was extracted from different flowering period libraries (early and full). The phenotypes of different flowering periods are shown in Figure 1. All the flower samples were collected on the 1st and 5th days of the flowering stage. Total RNA were used for the 454 pyrosequencing.

**Figure 2:**
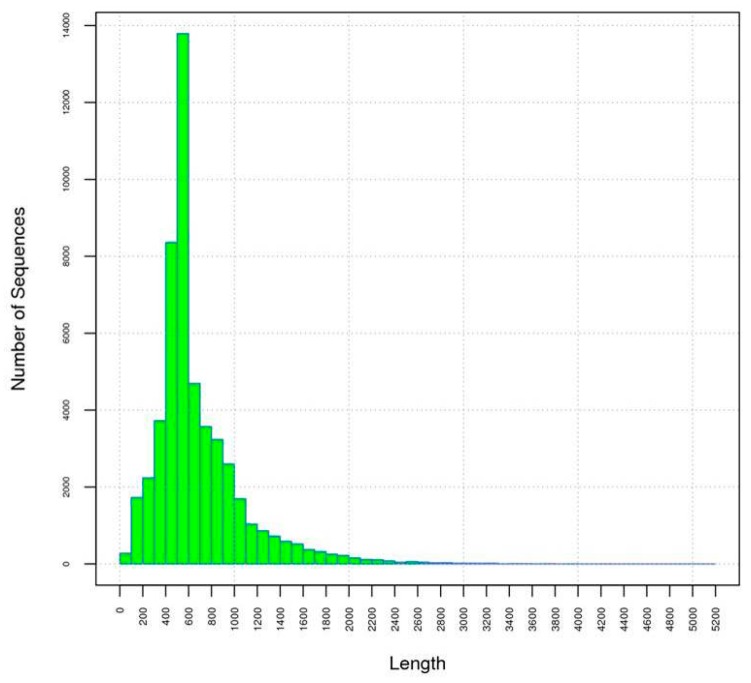
Length distribution of the safflower unigenes. The longest unigene was 5109 bp, and the average length of the unigenes was 679 bp.

The resulting sequencing data was analyzed using bioinformatics methods. A total of 583,440 and 567,884 raw reads were generated from early and full flowers by 454 sequencing, respectively (Table 1). After the removal of low quality reads, short reads (<50 bp), contaminating sequences and vector sequences by Tagdust (http://genome.gsc.riken.jp) [23] and Seqclean (http://compbio.dfci.harvard.edu) [24], 577,664 and 562,930 clean reads remained in the early and full libraries, respectively. The average read lengths of 427 and 436 bp, respectively, were used for assembling. Reads from two samples combined were assembled into 51,591 unigenes with an average length of 679 bp using the MIRA program [25] and CAP3 (http://seq.cs.iastate.edu). The longest read was 5109 bp. The length distribution of the reads is presented in Figure 2. The greatest number of sequences, 13,790, were between 501–600 bp in length.

**Table 1:** Summary of the sequence assembly after 454 sequencing.

	Early	Full
Raw reads	583,440	567,884
Low quality	50	67
Short reads (<50 bp)	796	928
Contamination sequences	2785	2694
Vector sequences	2145	1265
Clean reads	577,664	562,930
Average length	427 bp	436 bp

### 2.2. Comparison of Unigenes between Early and Full Flowering Stages

**Figure 3:**
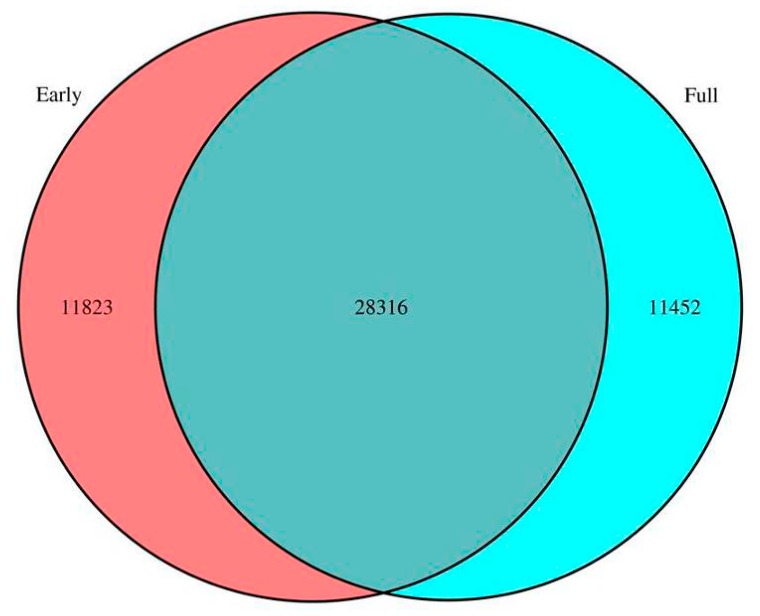
Safflower flowering gene expression statistics. The red represents genes that express in the early stage, and the blue represents genes that express in the full stage.

**Figure 4:**
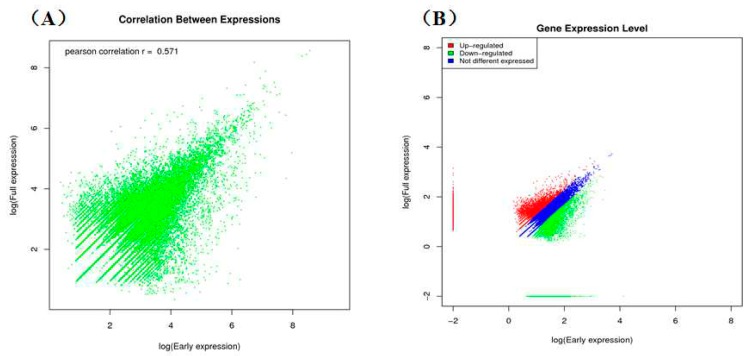
The scatter of expressed genes in the early and full flowering stages of safflower. The blue dots represent genes that are not differentially expressed, and the red and green dots represent up-regulated and down-regulated genes, respectively. (**A**) Correlation between expression in the early and full flowering stages of safflower; (**B**) Gene expression level in the early and full flowering stages of safflower.

Among the assembled unigenes, 40,139 were from the early flower group, including 11,823 unique unigenes, and 39,768 were from the full flower group, including 11,452 unique unigenes. In total, 28,316 unigenes were shared by the samples (Figure 3). The correlations between gene expression levels were measured using Pearson’s correlation values, which represent positive relative correlations as greater than zero and negative correlations as less than zero. The bitmap (scatter diagram) of correlations is shown in Figure 4A. Gene expression levels at different flowering stages were analyzed to estimate the differential expression between the two groups. With the threshold of “‘log2 ratio ≥ 1”, There were 18,043 up-regulated genes (red dots) and 16,421 down-regulated genes (green dots), with a total of 34,464 differentially expressed genes (DEGs) in the full flower stage when compared with the early stage. The number of unigenes with different expression levels is shown in Figure 4B. Additionally, 29.09% of the unigenes of the DEGs that differed by more than two fold in the early library and less than two fold in full library were up-regulated. 

### 2.3. Classification of Gene Ontology (GO) and Functional Annotation

**Figure 5:**
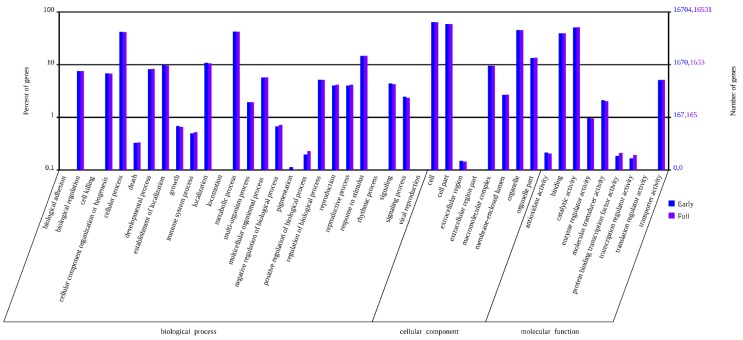
Gene ontology (GO) annotation of all safflower unigenes. There are 26 functional annotations in biological.

Gene ontology (GO) was used to functionally categorize annotated genes. In total, these unigenes were classified into 43 main functional groups, belonging to three main GO categories: biological process, cellular component and molecular function (Figure 5). The dominant GO terms in biological processes were grouped into cellular progress, metabolic progress and response to stimulus. Among cellular component, genes participated in cell, cell part, organelle and organelle part. Within the molecular function category, the assignments mostly involved binding and catalytic activity. There were a high number of genes from the of “cellular process”, “cell”, and “catalytic activity” and only a few genes from the categories of “pigmentation”, “extracellular region”, “protein binding transcription factor activity” and “transcription regulator activity”. More detailed information on the annotation is shown in Table S1.

**Figure 6:**
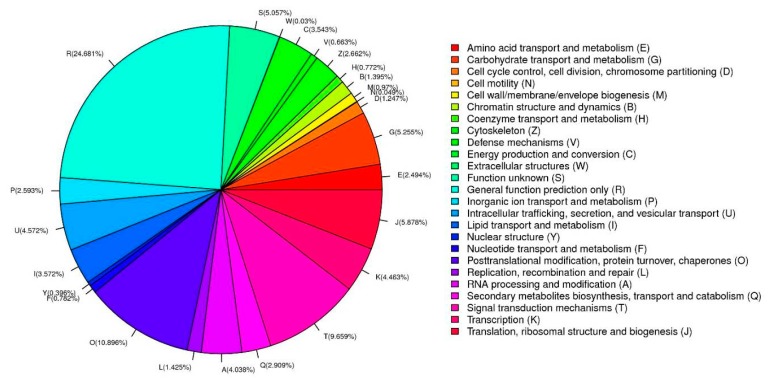
Clusters of orthologous groups (COG) classification. The safflower unigenes are classified into different functional groups based on the predicted proteins.

Clusters of orthologous groups (COGs) were utilized to further evaluate the functional annotation. There were 25 annotated functional COG categories (Table S2) of safflower unigenes (Figure 6), mostly related to metabolism. The five most represented categories were: “general function prediction only” (24.681%), “posttranslational modification, protein turnover and chaperones” (10.896%), “signal transduction mechanisms” (9.659%), “translation, ribosomal structure and biogenesis” (5.878%), and “carbohydrate transport and metabolism” (5.255%). The majority of genes were linked to transcription, folding, and molecular chaperone functions. In addition, the COG distribution of DEGs up-regulated and down-regulated is shown in Figures S1 and S2.

### 2.4. Kyoto Encyclopedia of Genes and Genomes (KEGG) Pathway Mapping and Analysis of DEGs

**Figure 7:**
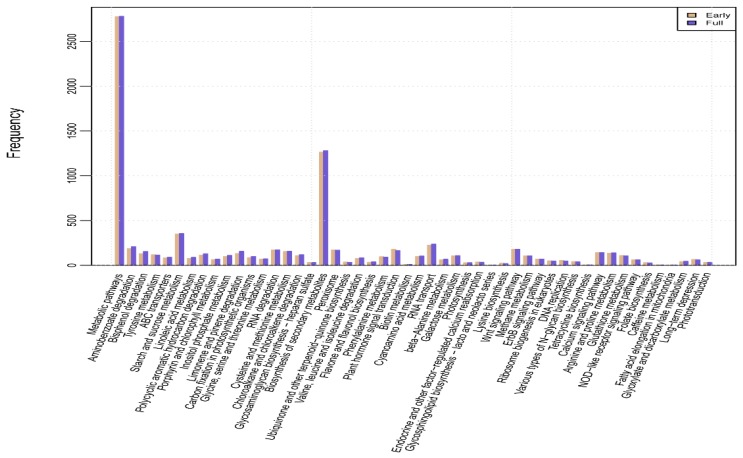
Kyoto Encyclopedia of Genes and Genomes (KEGG) pathways mapped in the safflower transcriptome. The 51,591 unigenes are annotated to 281 KEGG pathways.

The KEGG [26] database was used to analyze the metabolic or biological pathways and functions of gene products in the cells. KEGG annotation information provided a standardized pathway annotation of the safflower unigenes. To identify the biological pathways specific to safflower, we annotated and mapped 281 KEGG pathways for 51,591 unigenes (Figure 7). Based on an enrichment analysis of the DEGs and the KEGG annotation, a total of 189 pathways were estimated as up-regulated and 186 as down-regulated. Table 2 lists the pathways of the 15 most up-regulated and down-regulated DEGs. More detailed information regarding the pathways is shown in Table S3.

**Table 2:** The first 15 up- and down-regulated enriched pathways.

KEGG Pathway	Early	Full	*p* Value	*Q* Value	Pathway ID
up-regulated					
Bisphenol degradation	84	165	3.04 × 10^−8^	8.50 × 10^−6^	ko00363
Polycyclic aromatic hydrocarbon degradation	73	141	9.94 × 10^−8^	1.39 × 10^−5^	ko00624
Limonene and pinene degradation	83	171	5.17 × 10^−7^	4.83 × 10^−5^	ko00903
Aminobenzoate degradation	102	224	1.32 × 10^−6^	9.23 × 10^−5^	ko00627
Stilbenoid, diarylheptanoid and gingerol biosynthesis	69	142	4.35 × 10^−6^	2.44 × 10^−4^	ko00945
Valine, leucine and isoleucine degradation	44	97	0.00139643	0.0446	ko00280
Dioxin degradation	7	8	0.00143353	0.0446	ko00621
Phenylpropanoid biosynthesis	102	262	0.00205028	0.0574	ko00940
alpha-Linolenic acid metabolism	48	110	0.00236695	0.0602	ko00592
Cyanoamino acid metabolism	51	120	0.00345069	0.0805	ko00460
Fatty acid metabolism	49	118	0.00698693	0.15	ko00071
Benzoxazinoid biosynthesis	19	38	0.00912142	0.182	ko00402
Histidine metabolism	34	80	0.01505098	0.262	ko00340
Glycosylphosphatidylinositol(GPI)-anchor biosynthesis	39	95	0.01832198	0.262	ko00563
Flavone and flavonol biosynthesis	20	43	0.01950833	0.262	ko00944
down-regulated					
Plant-pathogen interaction	232	650	4.52 × 10^−8^	1.26 × 10^−5^	ko04626
Phagosome	642	2069	1.41 × 10^−7^	1.30 × 10^−5^	ko04145
Focal adhesion	607	1971	1.02 × 10^−6^	3.66 × 10^−5^	ko04510
Adherens junction	587	1923	5.39 × 10^−6^	9.99 × 10^−5^	ko04520
Tight junction	609	2015	1.33 × 10^−5^	2.32 × 10^−4^	ko04530
Monoterpenoid biosynthesis	13	20	0.00031602	4.62 × 10^−3^	ko00902
Apoptosis	47	117	0.00071701	9.97 × 10^−3^	ko04210
Biosynthesis of unsaturated fatty acids	74	210	0.0025018	0.0316	ko01040
Gap junction	45	120	0.00452067	0.0524	ko04540
SNARE interactions in vesicular transport	36	96	0.01032285	0.115	ko04130
Calcium signaling pathway	57	171	0.02407534	0.209	ko04020
Toll-like receptor signaling pathway	40	118	0.04043678	0.331	ko04620
Glycerophospholipid metabolism	51	157	0.04906638	0.39	ko00564
Sphingolipid metabolism	29	83	0.05075266	0.392	ko00600
Cell cycle	64	203	0.05448446	0.1	ko04110

### 2.5. Quantitative Real Time-PCR (qRT-PCR) Validation of 454 Pyrosequencing

**Figure 8:**
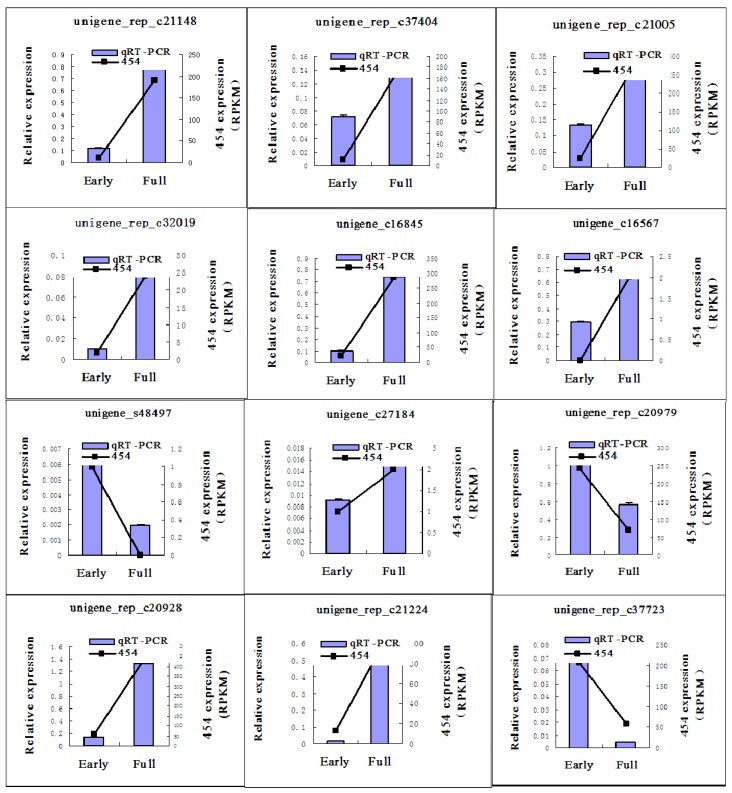
qRT-PCR validation of selected safflower unigenes. The qRT-PCR data are presented as the mean value of three repeats. “454” indicates the value of the unigene in 454 pyrosequencing. “Early” indicates the early flowering stage and “full” indicates the full flowering stage. Error bars indicate the standard deviation of the mean value of each stage.

To confirm the results of the 454 pyrosequencing, 12 unigenes were selected for qRT-PCR analysis. The relative expression levels of these unigenes were consistent with differential expression patterns in early and full flowering libraries (Figure 8). Unigene_s48497, unigene_rep_c20979 and unigene_rep_c37723 were confirmed as being more highly expressed at the early flowering stage compared with at the full flowering stage. Other annotated unigenes were confirmed as having their lowest expression levels during the early stage of flowering.

### 2.6. Analyses of the Flavonoid Biosynthesis Pathway and Putative Genes in the Transcriptome

**Figure 9:**
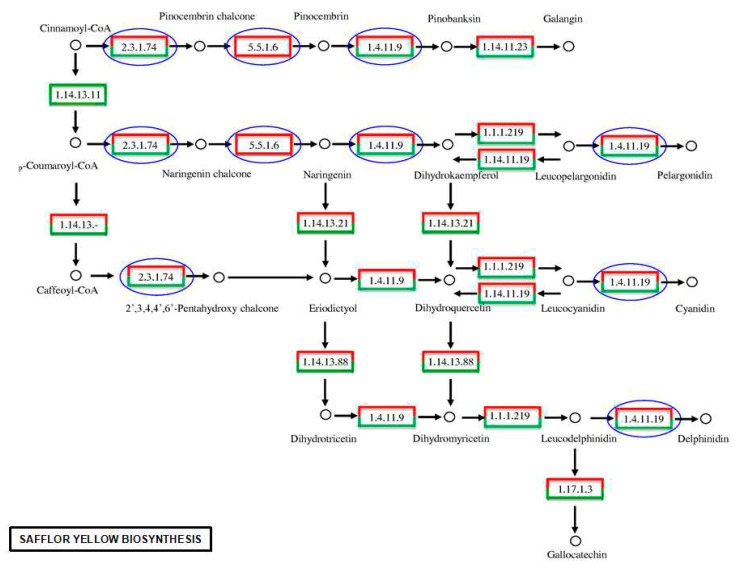
The metabolic pathways for safflor yellow biosynthesis. Blue circles indicate the important enzymes that were focused on in flavonoid biosynthesis. The red boxes represent up-regulation, and the green boxes represent down-regulation.

To identify flavonoid biosynthesis genes, we focused our analyses on KEGG pathways and safflower transcripts that appeared regulated in the two samples (Figure 9). In flavonoid biosynthesis, we were interested in the three key genes encoding flavonoid biosynthetic enzymes, chalcone synthase (CHS, EC 2.3.1.74), chalcone isomerase (CHI, EC 5.5.1.6) and anthocyanidin synthase (ANS, EC 1.14.11.19). The 22 unigenes, including those that were up-regulated and down-regulated, related to these genes are listed in Table 3. Of these, 9 were annotated as chalcone synthase (2 up-regulated and 4 down-regulated), 12 were annotated as anthocyanidin synthase (8 up-regulated and 4 down-regulated), and only 1 unigene (unigene_c27184) was annotated as chalcone isomerase.

**Table 3:** Unigenes predicted to be associated with flavonoid biosynthesis.

Gene	Code	unigene	DEG
chalcone synthase	EC 2.3.1.74	unigene_c16567 (2) unigene_rep_c22629 (1.07111231014049)	Up-regulated
		unigene_rep_c21289 (-3.00570328691034) unigene_c9855 (-2) unigene_rep_c33061 (-2) unigene_rep_c32553 (-2) unigene_rep_c30381 (-2) unigene_c13467 (-1.5809643864392) unigene_rep_c31854 (-1.29812149952523)	Down-regulated
chalcone isomerase	EC 5.5.1.6	unigene_c27184 (1.07111231014049)	Up-regulated
anthocyanidin synthase	EC 1.14.11.19	unigene_rep_c32431 (3.39304040502786) unigene_c5812 (2.8784672321981) unigene_rep_c20891 (2.13404356586695) unigene_c20381 (2) unigene_s42103 (2) unigene_c2096 (1.91910921669544) unigene_rep_c32424 (1.65607481086165) unigene_c9873 (1.65607481086165)	Up-regulated
		unigene_rep_c35725 (-2) unigene_s48497 (-2) unigene_rep_c33425 (-2) unigene_c20561 (-1.06639121360944)	Down-regulated

### 2.7. Validation and Expression Analysis of Putative Flavonoid Synthesis Genes from the Safflower Transcriptome

**Figure 10:**
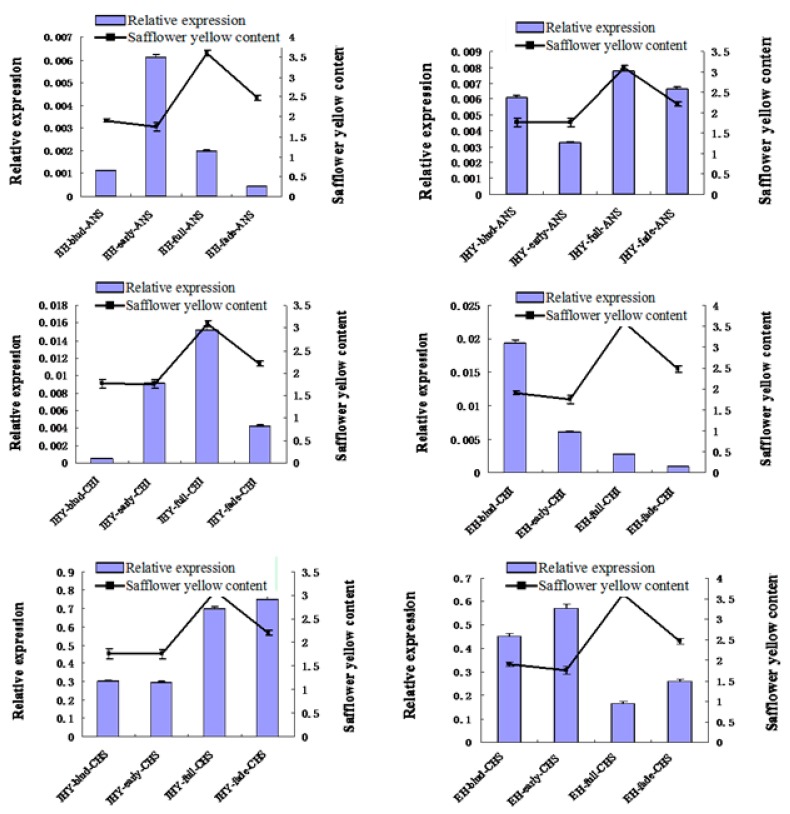
Relationships among the relative expression levels of the three genes and the content of Safflower yellow pigments during flower development in different varieties. HY, Jihongyou var.; EH, Jihongerhao var.; bud, early, full and fade indicate the flowering stages; CHS, chalcone synthase; CHI, chalcone isomerase; and ANS, anthocyanidin synthase. Data were obtained from three biological replicates of three independent experiments and are means ± SE (*n* = 3).

To validate that the unigenes that obtained from the safflower transcriptome and computational analysis were indeed expressed, qRT-PCR was performed to validate the expression levels of the flavonoid biosynthetic genes, CHS, CHI and ANS, at different flowering stages and in different varieties. In the qRT-PCR analysis (Figure 10), the selected genes showed distinctly different expression patterns in different varieties and during flower development. The CHS gene (unigene 27184) showed very highly expressed in all of the samples and the highest expression level was in the fading flower of Jihongyou, which was ~ 96 times greater than the highest expression of ANS (unigene_s48497) and 38 times greater than the highest expression of CHS (unigene_c16567). However, the lowest CHS expression level occurred in the full flowering stage of Jihongerhao, which was still greater than the expression of CHI and ANS. For the CHI gene, the highest expression level occurred in the full flowering stage of Jihongyou and the bud stage of Jihongerhao, while the lowest CHI expression level was in the bud of Jihongyou. The ANS gene had a lower expression, relative to CHS and CHI, in the four studied stages of flowering. SYP synthesis generally varied during flower development, with the highest amounts of SYP occurring in the full stage and lowest amounts in the bud or early stages, regardless of the variety.

### 2.8. Cloning and Sequence Analysis of the Full-Length cDNA of Key Gene in Flavonoid Synthesis

**Figure 11:**
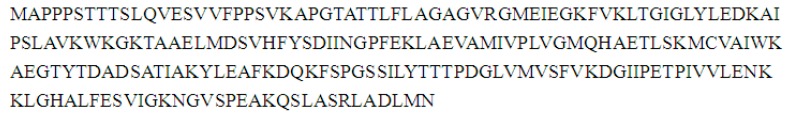
The amino acid sequence of chalcone isomerase (*CHI*) gene of safflower. It encoded 217 amino acids containing 654 bases.

**Figure 12:**
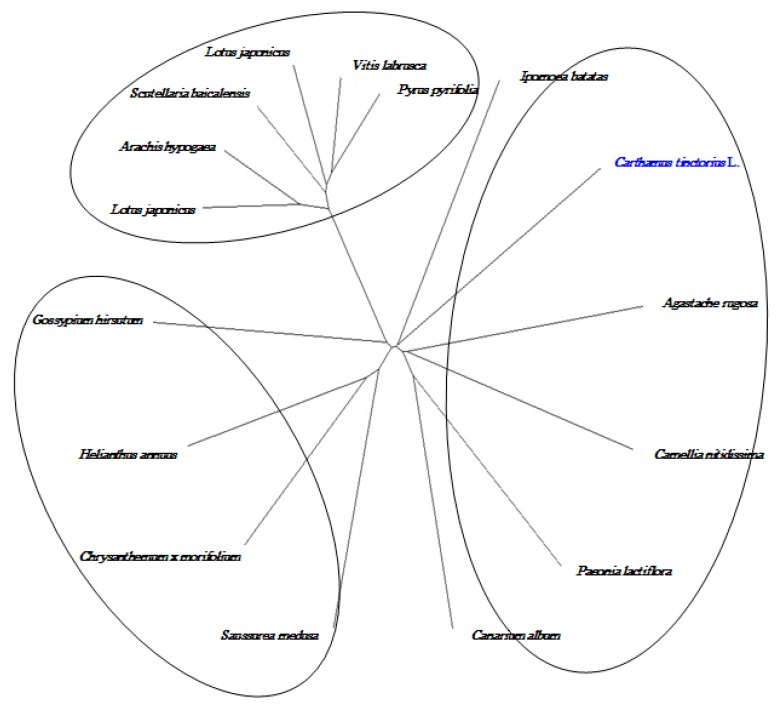
Phylogenetic tree of *CHI* gene from Safflower and other species. CHI gene in safflower has high homologywith *Ipomoea batatas*, *Agastache rugosa*, *Camellia nitidissima*, *Paeonia lactiflora* and *Canarium album.*

**Figure 13:**
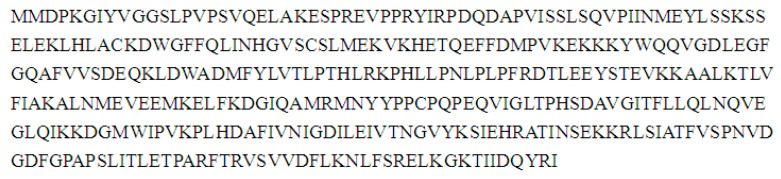
The amino acid sequence of anthocyanidin synthase (*ANS*) gene of safflower. It encoded a polypeptide of 349 amino acids.

**Figure 14:**
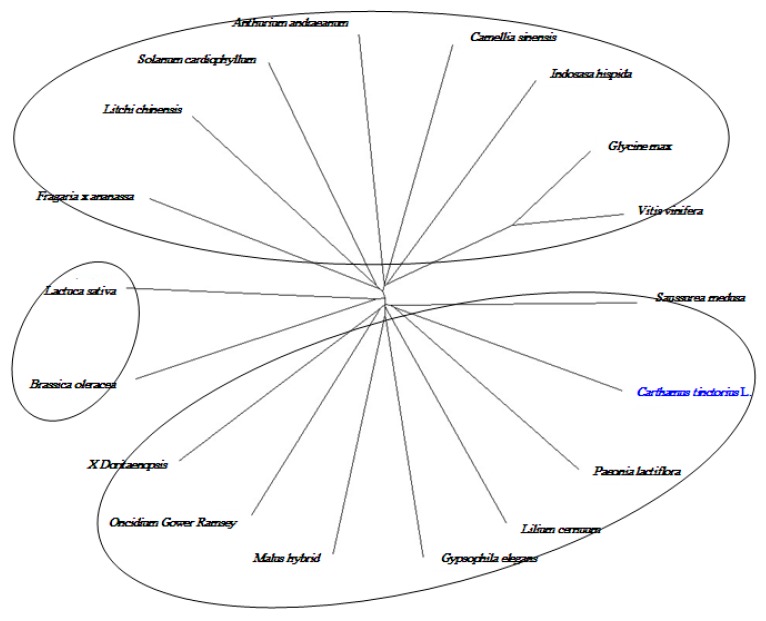
Phylogenetic tree of *ANS* gene from Safflower and other species. *ANS* of Safflower has higher homology with other plants.

To further confirm the results of the 454 pyrosequencing, full-length cDNA of chalcone isomerase (CHI) and anthocyanidin synthase (ANS) were cloned from safflower petal by RACE method according to fragment of the transcriptome, the 1161 bp CHI cDNA contained a 654 open reading frame (ORF) that encoded 217 amino acids (Figure 11) and phylogenetic tree showed that CHI gene in safflower has high homology (Figure 12) with other species, *Ipomoea batatas*, *Agastache rugosa*, *Camellia nitidissima*, *Paeonia lactiflora* and *Canarium album* were separated into a large groups. The putative protein of CHI gene showed predicted molecular weight of 23.14 kD with a theoretical pI of 5.67, containing typical AATAA tail signal sequence and Poly(A). By similar method, the cDNA sequence of ANS gene which was 1226 bp and included a whole open reading frame of 1050 bp, encoding a polypeptide of 349 amino acids (Figure 13). A phylogenetic tree was drawn to investigate the evolutionary relationship contrasting to previously reported ANSs from other plants and found higher homology between them (Figure 14). The conserved structural domain analysis showed that ANS gene had thetypical functional domains of ANS protein, containing 2-oxoglutarate and iron ion combination sites.

## 3. Discussion

Safflower is an economically important traditional Chinese medicinal crop, which is well-adapted to growth in arid environments [27]. It has significant economic value and pharmacological effects, based on the amount of safflor yellow in curing. High-throughput sequencing technology has been widely used in various plants to obtain transcript coverage even without a reference genome. 454 sequencing is a reasonably low cost [28] transcriptome profiling method, and its novel and efficient high throughput approach has been used on the olive [29], *Leymus chinensis* [21], orchids [30], *Podophyllum hexandrum* [31], plum [32], *Lonicera japonica* Thunb. [33] and *Vicia faba* L. [34]. Here, 454 sequencing was applied to the safflower transcriptome for the first time, to discover the important genes in flavonoid biosynthesis, which may be related to the synthesis of SYPs during different flower developmental stages. We used this method to generate transcripts and analyzed gene expression and secondary metabolic pathways, demonstrating that 454 sequencing is an important tool in gene discovery, and in the study of gene expression, genetic markers and genomics.

Although a previous high-throughput sequencing platform, Solexa/Illumina [9,20], had been applied in safflower, the objective of the present study was to analyze differences in samples based on the database sequencing. The 454 sequencing produced large numbers of clean sequencing reads that were used to *de novo* assemble and functionally annotate unigenes from different flowering stages. We obtained 1,140,594 clean reads (577,664 and 562,930 clean reads in early and full flowering stage samples, respectively) which were assembled into 40,139 unigenes using *de novo* assembly. The results contained reads that corresponded to those obtained with 454 pyrosequencing in *P. hexandrum* [35], *Lonicera japonica* Thunb. [36] and *Vicia faba* L. [37]. We mapped 51,591 unigenes to 281 KEGG pathways, which and classified into 43 main functional groups. This data provides a foundation for further studies on secondary metabolism in safflower.

In this study, gene expression levels were compared with the Safflor yellow contents in different varieties and flowering. In the full stage of Jihongyou, the expression levels of CHS, CHI and ANS were higher than in the other flowering periods. While in Jihongerhao, the lowest expression levels of the three genes occurred when the pigment levels peaked during the full fade. Our results showed that the increase or decrease in CHS, CHI and ANS expression levels was mainly related to the accumulation of Safflor yellow, but the expression levels and contents of pigments varied in different varieties, suggesting further research on the mechanism of CHS, CHI and ANS in the flavonoid synthesis pathway is required.

CHS is the first pivotal enzyme in the flavonoid synthesis pathway [38], and it participates in a series of physiological and biochemical reactions, such as flavonoid biosynthesis, pigment synthesis and resistance. CHS expression levels are different in various plants, tissues and developmental stages. In this study, the expression level of CHS is higher than that of CHI and ANS during flower development, demonstrating that the CHS gene may be important for safflower flavonoid synthesis. This result is consistent with that of Huang [39]. The overexpression of CHI can increase the flavonoids content [22] and CHI expression in safflower shows its regulation is related to flowering. The trends in gene expression are identical to those found in white narcissus [28], indicating that CHI may be involved in the synthesis of flower colors and pigments. 

An important medicinal aspect of safflower is its tubular flowers [2], and HSYA, and flavonoid compounds, are extracted from safflower petals. Another object of this study was to discover genes related to flavonoid biosynthesis. Using the KEGG database, we mapped unigenes to the flavonoid biosynthesis pathway and identified multiple genes from this pathway. We focus on important enzymes, CHS, CHI and ANS, that contribute to flavonoid compound synthesis according to the previous reports [12,14]. The expression analysis of these genes using qRT-PCR indicated that their relative expression levels in different samples high expression of CHS, demonstrating that the CHS gene is important in safflower flavonoid metabolic synthesis, which is consistent with a previous study [9]. 

## 4. Experimental Section

### 4.1. Sample Preparation and RNA Extraction

Safflower seeds were purchased from XinJiang (Honghua Yuan Co., LTD), China, and grown in 16 cm diameter pots under greenhouse conditions of 26 °C (day) and 20 °C (night), 80% relative humidity; light for 16 h (intensity of illumination at a constant 30,000 lx) and dark for 8 h [20]. When blooming, the early stage occurred on the first day after flowering and the full stage occurred on the fifth day (Figure 1). The petals of early and full safflowers were frozen quickly in liquid nitrogen and stored at −80°C. Total RNA was isolated from individual petals using Trizol (Invitrogen, Carlsbad, CA, USA) according to the manufacturer’s protocol for cDNA library construction by 454 sequencing. The RNA quality and quantity were determined using gel electrophoresis and a NanoDrop 2000 (Thermo Scientific, Wilmington, DE, USA), respectively.

### 4.2. De Novo Assembly and Sequence Analysis 

High quality total RNA was prepared for 454 sequencing using a GS-FLX sequencer (Roche, Basel, Switzerland). The clean reads were obtained by deleting raw data, including low quality reads, short reads (<50 bp), adaptor reads, polluted reads, and hairpin structural reads, and were assembled into unigenes utilizing the MIRA program [25]. Then, the unigenes from the two samples were used for future analyses.

### 4.3. Comparative Analysis between Two Samples

The expression abundance in the sample is shown by the number of reads mapped onto unigenes in the sample, as represented by normalized reads, such as Reads Per Million reads (RPM) and Reads Per Kilo bases per Million reads (RPKM) [38], which were used to compare the relative expression levels of the two samples in further analyses. DEGs were deemed to be unigenes with thresholds of “log2 ratio ≥ 1” and “false discovery rate < 0.001” for sequence counts across the early and full samples. Subsequently, a functional enrichment analysis of KEGG and metabolic pathways was performed on the DEGs.

### 4.4. Functional Annotation and Analysis of Pathway Enrichment

To predict the protein of the highest similarity to preset unigenes, functional annotations of the genes and analyses of pathway enrichment for the unigenes were carried out via the following databases: Nr (http://www.ncbi.nlm.nih.gov) and Nt (http://www.ncbi.nlm.nih.gov/). Annotations were performed by blast (blastn and blastx). Additionally, blastx was used to reveal functional annotations in SwissProt (http://www.ebi.ac.uk/uniprot/) and COG/KOG (http://www.ncbi.nlm.nih.gov/COG/). GO (http://www.geneontology.org/) annotations and GO functional classifications were determined using blast2GO [40] and WEGO software [41], respectively. The KEGG pathways (http://www.genome.jp/kegg/) were annotated using blastx and GenMAPP 2.1 (http://www.genmapp.org/) [42]. 

### 4.5. qRT-PCR Validation of 454 Pyrosequencing

To validate the accuracy of 454 pyrosequencing in different flowering stages, the expression levels of 12 selected date genes were determined using qRT-PCR. Total RNA was extracted from safflower using the Super RT Kit (Takara, Japan). The 18s rRNA gene was used as an internal control [43], and the qRT-PCR was performed according to the SYBR^®^ Premix Ex Taq protocol (Takara, Japan) using a Stratagene Mx3000P instrument (Agilent, Palo Alto, CA, USA). The final volume of the qRT-PCR reaction was 20 μL, including 2 μL of cDNA, 10 μL SYBR Premix Ex Taq (Tli RNaseH Plus), 0.4 μL ROX Reference Dye II (50×), 0.4 forward and reverse primers (10 mM) and 6.8 μL ddH_2_O. The relative quantification of gene expression was computed using the 2^−Δ*C*t^ method.

### 4.6. Analysis of Putative Flavonoid Synthesis Genes in the Safflower Transcriptome

The flavonoid biosynthetic pathway was mapped in the KEGG database using the relative expression levels in different samples. Putative genes involved in the flavonoid biosynthetic pathway were used as query to search the NCBI databases, and unigenes of the highest similarity with other species were selected to confirm the sequencing results.

### 4.7. qRT-PCR Analysis and the Isolation of Important Genes in Flavonoid Synthesis

An expression analysis of the selected putative genes in flavonoid synthesis were determined using qRT-PCR. Total RNA was extracted from tissue samples, including different varieties (Jihongerhao and Jihongyou) and flowering stages (bud, early, full and faded), approximately 1 μg of DNaseI-treated total RNA was converted into single-stranded cDNA using a Super RT Kit (Takara, Japan). Gene specific primers were designed to amplify three genes of interest from the transcriptome, and RT-PCR was performed for preliminary verification before the qRT-PCR analysis. The 18s rRNA gene was used as an internal control, and the qRT-PCR was performed according to the protocol of SYBR^®^ Premix Ex Taq (Takara, Japan) using a Stratagene Mx3000P instrument (Agilent). The final volume of the qRT-PCR reaction was 20 μL, including 2 μL of cDNA, 10 μL SYBR Premix Ex Taq (Tli RNaseH Plus), 0.4 μL ROX Reference Dye II (50×), 0.4 forward and reverse primers (10 mM) and 6.8 μL ddH_2_O. The relative quantification of gene expression was computed using the 2^−Δ*C*t^ method [29]. The full-length cDNAs of the two genes were isolated and analyzed from Safflower petals using 5′ and 3′ RACE methods in the SMART RACE Amplification kit (Clontech, Japan). The RACE fragments were amplified by specific primers, ligated into the pEASY-T1 vector (TranGene Biotech, Beijing, China) and sequenced. Then, the RACE fragments were used for subsequent verifications and analyses of full length cDNAs.

## 5. Conclusions

In this report, a comprehensive transcriptome database from different safflower flowering stages was obtained using 454 pyrosequencing and produced 577,664 and 562,930 clean reads from the early and full flowering libraries, respectively. The safflower transcriptome provided 34,464 DEGs and a large number of unigenes mapped to 281 KEGG pathways. The qRT-PCR analysis of selected unigenes indicated that the 454 sequencing was accurate. We annotated a large number of genes involved in safflor yellow biosynthesis and studied the expression of putative genes related to safflor yellow synthesis. The data from this study will enrich our knowledge of safflower and provide a theoretical foundation for functional studies of SYP-related genes using transgenic technologies. Full-length cDNA of chalcone isomerase (CHI) and anthocyanidin synthase (ANS) were cloned from safflower petals by the RACE method.

## Supplementary Materials

Supplementary materials can be found at http://www.mdpi.com/1422-0067/16/10/25657/s1.
