# Supplementary material for: De Novo Sequencing and Analysis of the Safflower Transcriptome to Discover Putative Genes Associated with Safflor Yellow in Carthamus tinctorius L
Source: Int J Mol Sci. 2015 Oct 26;16(10):25657–77. doi: 10.3390/ijms161025657 (PMC4632820; doi:10.3390/ijms161025657)
Supplement: Supplementary file 1 [file ijms-16-25657-s001.zip › ijms-100681-Figures S1 and S2.pdf]

Supplementary Information

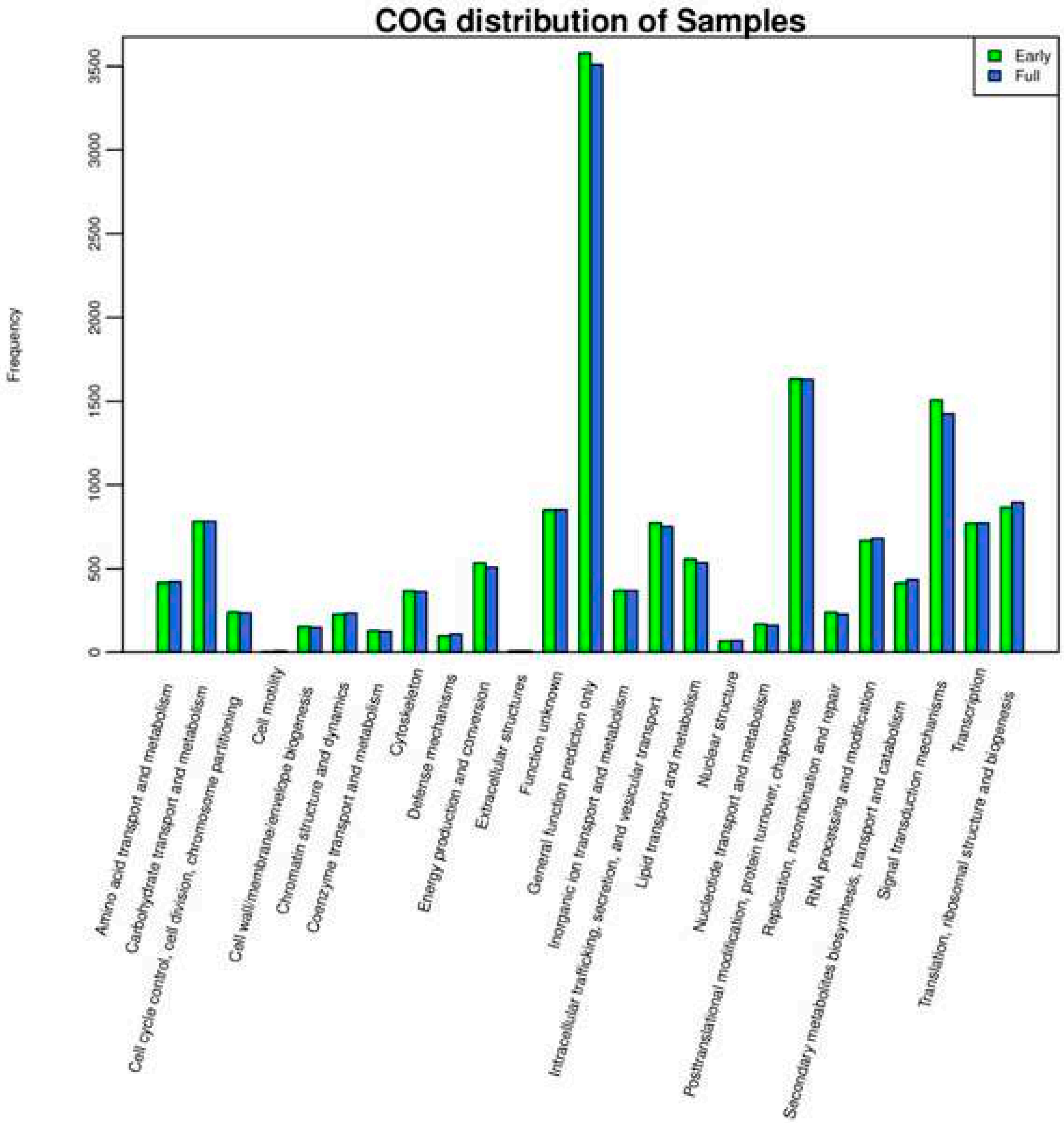

Figure S1. COG distribution of safflower DEGs.

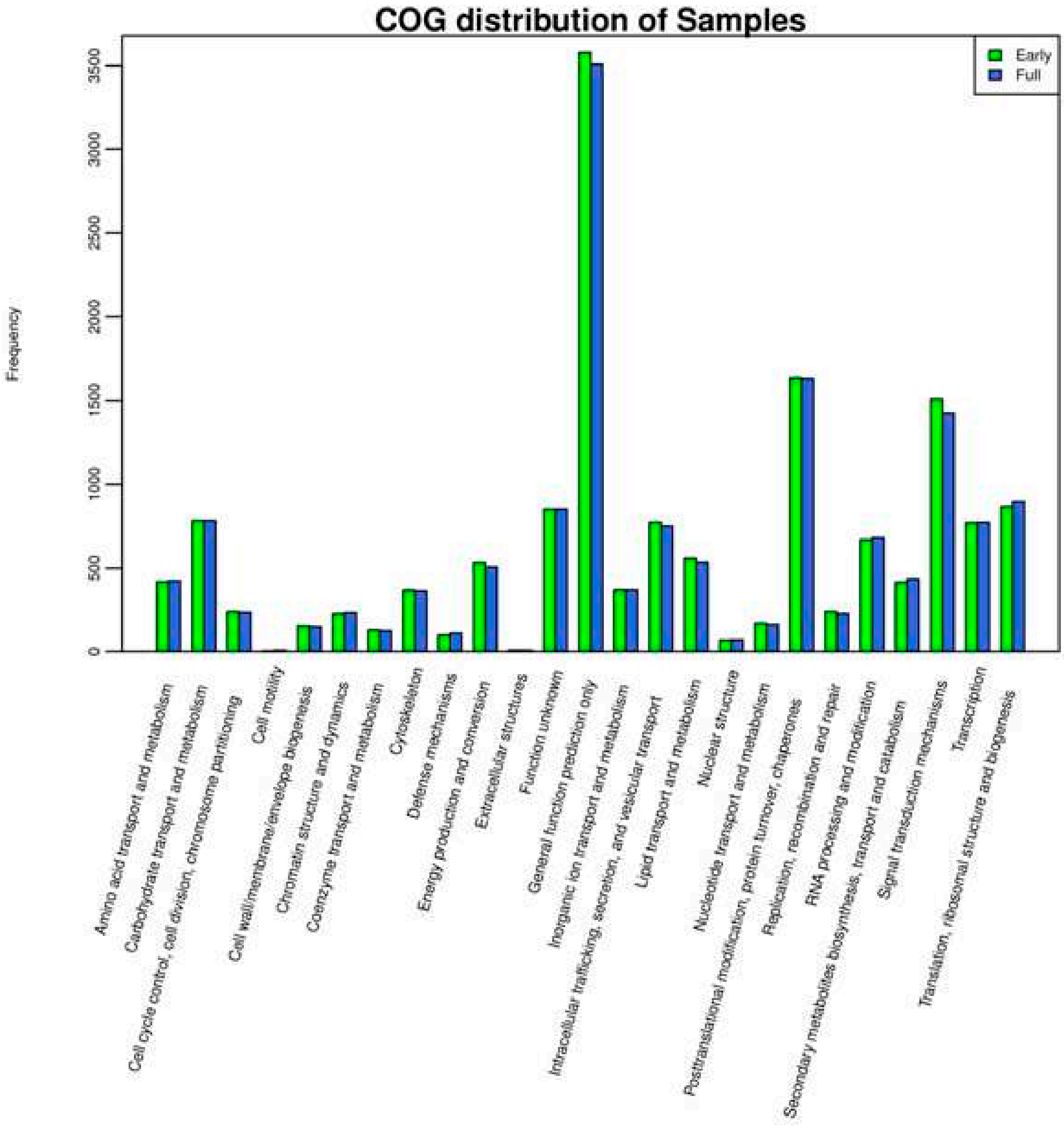

Figure S2. COG distribution of safflower samples.
